# *“cu-coo”*: Can You Recognize My Stepparents? – A Study of Host-Specific Male Call Divergence in the Common Cuckoo

**DOI:** 10.1371/journal.pone.0090468

**Published:** 2014-03-06

**Authors:** Won-Ju Jung, Jin-Won Lee, Jeong-Chil Yoo

**Affiliations:** Korea Institute of Ornithology & Department of Biology, Kyung Hee University, Seoul, Republic of Korea; Virginia Tech Virginia, United States of America

## Abstract

The presence of multiple host-specific races in the common cuckoo *Cuculus canorus* has long been recognized as an evolutionary enigma but how this genetic divergence could be maintained is still equivocal. Some recent studies supported biparental genetic contribution in maintaining the host-races, implying the necessity that they should recognize and mate assortatively with those who belong to the same host-race. One potential mechanism to accomplish this is that males may produce distinctive calls according to host-specific lineages. In order to test this hypothesis, we carried out a comparative study for male cuckoo calls recorded from three distant populations, where two populations share a same host species while the other parasitizes a different host species. Populations with similar habitat structures, maintaining comparable distance interval (*ca.* 150 km) between neighboring ones, were selected so as to minimize any other causes of vocal differentiation except the pattern of host use. By comparing the vocal characteristics of male cuckoos at the level of individual as well as population, we found that individual males indeed produced different calls in terms of spectral and temporal features. However, these differences disappeared when we compared the calls at the population level according to host species and geographic location. In conclusion, it seems unlikely for the cuckoos to identify the stepparent of male cuckoos based solely on the vocal characteristics, although they may be able to use this cue for individual recognition. Future studies including detailed morphological and genetic comparisons will be worthwhile to further elucidate this issue.

## Introduction

As a model system for the study of reciprocal natural selection, brood parasitism by the common cuckoo *Cuculus canorus* has attracted a lot of research interests over a long period (e.g., [1]–[7]). It is now said that cuckoos parasitize over 100 different host species across their range but at the same time they specialize on a particular host species at a local level [6], [8]–[11]. Due to cuckoo parasitism inflicting acute fitness costs to hosts, this specific interaction involving high selection pressures cause an evolutionary arms race between the parasites and hosts; the hosts develop a defense strategy against cuckoo parasitism and the parasites counter-adapt to overcome this host defense [6]. As a key fitness component, genetically determined phenotypic adaptation such as egg color mimicry is at the core of this race [12], [13]. Hosts recognize and reject conspicuous parasitic eggs, which provoke cuckoos to mimic egg colors and spotting patterns of host species. Furthermore, not only phenotypic adaptation but innate behavioral adaptation was also reported in this race. For example, Davies et al. [14] demonstrated that cuckoo chicks possess an innate pre-tuning alarm call response so that they react specifically to the alarm call of stepparents on which their biological mothers specialize. All these host-specific traits are genetically determined, which may indicate that the cuckoos might be composed of multiple host-specific races with distinct genetic profiles [1], [9].

As an evolutionary puzzle, an intriguing question that naturally arises is how multiple host-specific races coexist within a species. In other words, how genetic divergence among the cuckoo host-races maintains without speciation in the cuckoos? A conventional answer that was first supported by some field and genetic studies is that each female lineage may specialize on a particular host species over an evolutionary time scale (‘the gentes hypothesis’) [15]–[17]. More specifically, if the genes for host-specific traits are located on the female specific W chromosome (in birds, females are heterogametic in sex chromosome), then female-oriented host-specific races can be maintained without disturbance by the genetic contribution of males [16], [17]. Furthermore, if male cuckoos choose a mate irrespective of her host-specificity, then gene flow between the cuckoo host-races could occur and thus the cuckoos could remain as one species. However, increasing recent evidence has challenged this traditional view. First of all, it seems almost unlikely that all the genes controlling host-specific traits such as egg colors and nestling behavior are located on W chromosome or other sex-linked loci. This is because the W chromosome turned out to be so small that it contains few functional genes [18], and it is also difficult to explain the fact that innate behavioral adaptations such as host-specific alarm call response are observed in both sexes of cuckoo chicks [14]. Therefore, considering the sophisticated host-specific adaptation shown by both sexes of the cuckoo, the gentes hypothesis sounds plausible at first but now it seems to need further evidence to be confirmed concretely.

Recent empirical and theoretical studies have supported the alternative view that not only females but males also may belong to a host-specific race and thus the cuckoo may be a complex of cryptic species (‘the cryptic species hypothesis’, [19]). Fossøy et al. [20] showed that biparentally inherited microsatellite DNA as well as maternally inherited mitochondria DNA diverged significantly among the cuckoo host-races at a local scale (ca. 10 km^2^). This implies that male and female cuckoos mate assortatively according to preferable host species, being a potential support for this alternative view. With the simulation modelling approach, Krüger & Kolss [21] also showed theorically that a coevolutionary arms race between the parasites and hosts may result in male cuckoos having host fidelity, which in turn causes genetic divergence among the cuckoo host-races. Furthermore, as mentioned above, the genes for innate behavioral adaptation should be inherited biparentally because it occurs in both sexes [14], confirming again the necessity of paternal contribution in the inheritance of host-specific traits.

Under the scenario of the cryptic species hypothesis, they should mate selectively with partners who belong to the same host-races so that they can avoid the collapse of this finely-tuned host-specific adaptation and thus keep genetic host-specific races. As a potential mechanism to this, vocalizations may be one of the first cues to be proposed in birds. In line with this hypothesis, Fuisz and de Kort [22] showed that call structures differed significantly among male cuckoos occupying different types of habitats, with which they proposed the possibility of assortative mating according to habitat types. Because both sexes of the cuckoo are known to be imprinted on where they grew up, and they appear to parasitize one predominant host in each habitat they occupy [23], habitat-specific male call divergence may play a potential role in maintaining genetic host-specific adaptation.

However, the evidence suggest that female cuckoos seem to selectively exploit one host species even though they could potentially use multiple species within a certain habitat [24], indicating that cuckoos seem to have a fidelity to not only a specific habitat type but also a specific host species within a habitat. This situation may imply the need of a more direct prediction; that is, the characteristics of male calls should diverge according to host races rather than habitat types in order to maintain host-specific genetic adaptation. So far, however, experimental studies testing this prediction are still lacking, despite its importance in the evolutionary study of avian brood parasitism. This may be partly due to practical difficulites in catching and housing the sufficient number of cuckoos in the field. A comparative approach would be an alternative to test the prediction while escaping such difficulties. In this study, the vocal characteristics of male cuckoos that exploit different host species were examined. We first investigated if male cuckoos indeed produce different calls at the individual level and then analyzed how these differences are associated with host specificity of male cuckoos at the population level.

## Methods

### Ethics statement

Field studies were carried out in accordance with relevant national and international guidelines and did not involve endangered or protected species. Male cuckoo calls were recorded at three geographically distant sites which are located in Chungcheongnam-do (36°27′N, 127°07′E), Jeollanam-do (34°48′N, 126°22′E) and Jeju-do (33°31′N, 126°32′E), respectively and no specific permissions were required for these locations and activities.

### Study system

**Figure 1:**
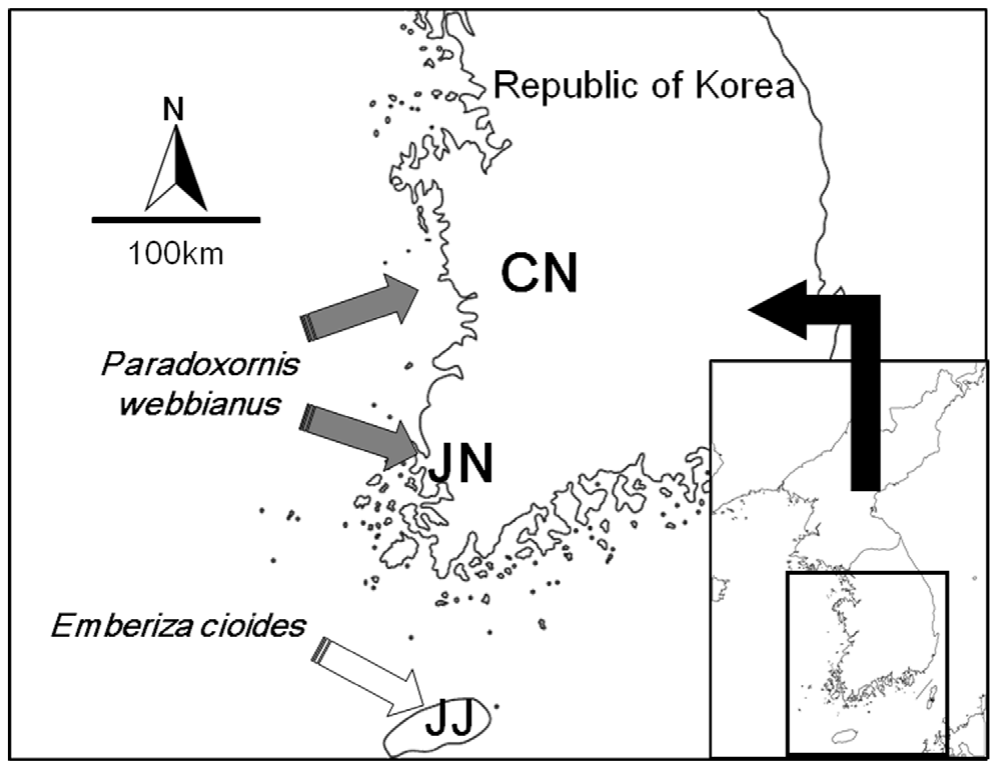
Location of study sites and the main hosts of the common cuckoos *Cuculus canorus*. Two sites are located along the mainland of Korea (Chungcheongnam-do (CN; 36°27′N, 127°07′E), Jeollanam-do (JN; 34°48′N, 126°22′E)) and one site is on an island, Jeju-do (JJ; 33°31′N, 126°32′E). The vinous-throated parrotbill *Paradoxornis webbianus* is known to be the main host species in the mainland of Korea while this species does not inhabit JJ at all. The meadow bunting *Emberiza cioides* is known to be a potential primary host on JJ.

Fieldwork was conducted at three geographically distant sites located in Chungcheongnam-do, Jeollanam-do, and Jeju-do (hereafter, “CN”, “JN”, “JJ”, respectively; Fig. 1); each site covers about 500 km^2^. CN and JN are located along the mainland of Korea and JJ is the largest island (1,848 km^2^) of Korea located at the south of the Korean peninsula. Each of the neighboring populations are about 150 km away from each other, which let us control the effect of geographic distance on call divergence. The habitat structures of three study sites were more or less similar in that they were open fields surrounded with mountains and forests, although vegetation types might be dissimilar due to latitudinal difference. Interestingly, cuckoos in JJ are clearly different in that they exploit a different host species from the other two populations. In the mainland of Korea, the primary host parasitized by the cuckoos is the vinous-throated parrotbill, *Paradoxornis webbianus* [25]. As secondary host species, the daurian redstart *Phoenicurus ochruros* and the stone chat *Saxicola torquatus* are also parasitized by the cuckoos, albeit with low frequency. The eggs of all of these host species have blue lines of background color and the cuckoos parasitizing these species in mainland Korea mimic their eggs very well. However, these species do not inhabit JJ or at least do not breed in JJ at all [26]; instead, anecdotal observations suggest that the meadow bunting *Emberiza cioides* which lays grayish white eggs with black lines may be a potential primary host in JJ [26]. The meadow bunting is known as a strong rejecter [27], which thus imply that the cuckoo host-race using this species should mimic their eggs well in order to overcome host defence. Therefore, it is highly unlikely that the cuckoo-race laying blue eggs in mainland Korea successfully parasitize the meadow bunting's nests which contain grayish white eggs in JJ. This circumstance evidently indicates that whichever species cuckoos in JJ parasitize, they form a distinct host-specific race from those in CN and JN of the mainland of Korea. Therefore, these situations may provide a unique chance to elucidate male call divergence with respect to host use while controlling geographic cline in call variation.

### Call records and analyses

**Figure 2:**
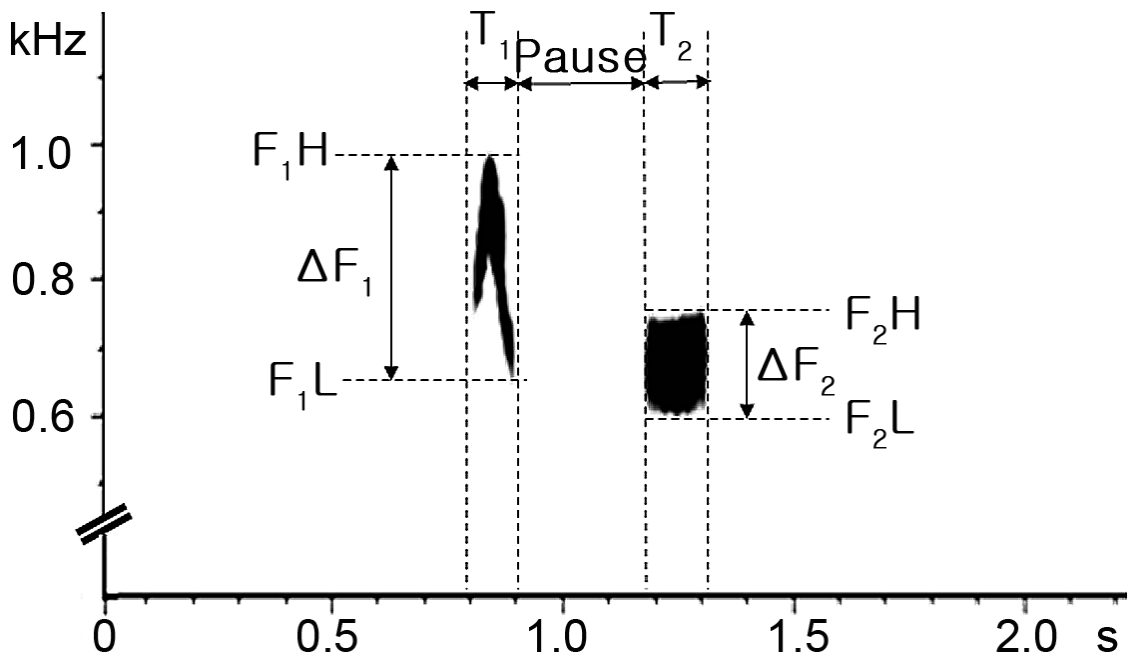
Spectrogram of a male cuckoo call with 9 measurable variables. T_1_ and T_2_ denote the duration of syllables 1 and 2, respectively; a pause represents the temporal gap between syllables 1 and 2; F_1_H and F_2_H indicate the highest frequency of syllables 1 and 2, respectively; F_1_L and F_2_L denote the lowest frequency of syllable 2. ΔF_1_ and ΔF_2_ represent the difference between the highest and the lowest frequencies of syllables 1 and 2, respectively. PF1 and PF2 are the peak frequencies of syllables 1 and 2, respectively and ΔPF denotes the difference between the peak frequencies of syllables 1 and 2.

Using a Zoom H1 recorder (ZOOM) with a directional microphone, ordinary male cuckoo calls were recorded at three locations (CN, JN, JJ) between May and June in 2012. In order to avoid duplicated recording of the same individual, we regularly changes the study site by driving to new locations, never again visiting a place where we conducted recordings. Once we spotted a calling male, we tried to approach him as closely as possible in order to record his calls clearly. Occasionally, we used playback to check for the presence of cuckoos where no cuckoo calls were heard, stopping playback as soon as a male was located to prevent playback calls affecting its behavior. Recordings were digitized with FairStars Audio Converter (v 1.97), at 16 bits at a sample rate of 44.1 kHz to a PC. To remove background noise, the digitized sound files were filtered with band-pass filters in Sound Analysis Pro (SAP, [28]). A range of calls which were at a similar level of amplitude were extracted from each individual to minimize measurement errors caused by variable amplitude. Then, because ordinary male calls are individually highly repeatable (see below and results), we randomly chose one call per individual from this range of individual calls for further analysis. Referring Fuisz and de Kort [22], we obtained 9 variables and 3 derived parameters from this call in SAP (Fig. 2): duration time (T_1_, T_2_), interval duration time (pause), highest · lowest frequencies (F_1_H, F_1_L, F_2_H, F_2_L) of 1^st^ syllable and 2^nd^ syllable, peak frequencies (PF_1_, PF_2_), the differences between maximum and minimum frequency (ΔF_1_, ΔF_2_) and the gap of peak frequency between 1^st^ and 2^nd^ syllable (ΔPF). The frequency parameters were averaged across a succession of narrow and overlapping time windows (FFT data window  =  10 ms, advance window  =  1 ms). All parameters were taken by one observer to avoid interobserver bias.

### Statistical analyses

The consistency of male cuckoo calls was measured by calculating the repeatability (r) of each parameter of the calls based on repeated measure of individual calls and an analysis of variance [29], for which we selected 3 males per site and 5 different calls per individual; in total 9 individuals with 45 different calls were used.

To account for correlations among call parameters and avoid multiple testing, call features extracted by SAP were examined with a Principal Component Analysis (PCA). We retained principal components with eigenvalues greater than 1 after varimax rotation by the Kaiser criterion. Then we conducted an ANOVA to determine geographical differences of principal component scores among the three populations. In addition, we tried to combine the data according to the pattern of host utilization (JJ *vs.* CN+JN) and then compared their component score using Welch's t-test [30]. All statistical analyses were done with R version 2.14.1 [31].

## Results

### Individual call variation and consistency

**Figure 3:**
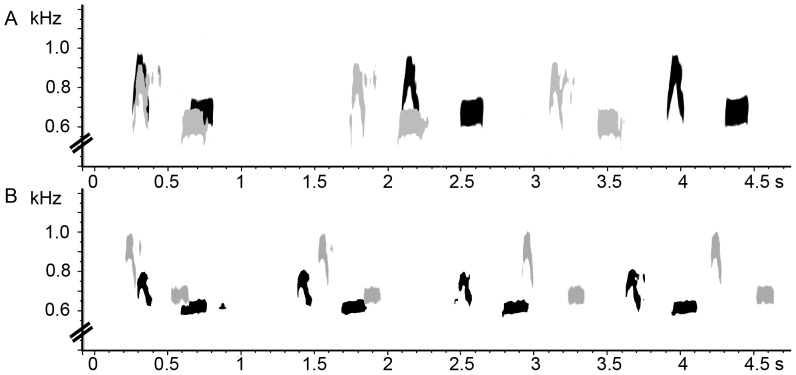
Spectrograms of male cuckoo calls illustrating between-individual variation and within-individual consistency. Ordinary calls of male cuckoos differ individually, showing consistent differences in time duration (A) and frequency range (B) of their calls. Colors of spectrograms in each panel represent different individuals.

In total, the ordinary calls of 24, 22 and 22 different males were recorded in CN, JN and JJ, respectively. Calling male cuckoos typically produced clearly distinct two-syllable calls, in which the first syllable was louder and higher pitched with the maximum frequency below 1 kHz (Fig. 2). Overall, there was a broad range of variation in each call parameter (Table 1), which may suggest that individual calls differ from one another. The repeatability test using 45 calls from 9 individuals revealed that most call parameters indeed varied and these differences resulted from between-individual variation rather than within-individual, indicating that each male produced a highly consistent call (Table 1, Fig. 3).

**Table 1:** The range of 9 call parameters and its consistency measured by calculating repeatability (r).

Parameters	Mean ± s.d.^a^	Range (Min–Max)^a^	r^b^	F^b^	P^b^
T_1_	85±12.8 ms	62–118 ms	0.72	F_8,38_ = 14.29	p<0.001
pause	236±29.3 ms	175–310 ms	0.85	F_8,38_ = 29.90	p<0.001
T_2_	148±20.1 ms	107–219 ms	0.58	F_8,38_ = 8.12	p<0.001
PF_1_	792±32.6 Hz	715–883 Hz	0.58	F_8,38_ = 8.15	p<0.001
PF_2_	642±26.4 Hz	595–708 Hz	0.94	F_8,38_ = 81.3	p<0.001
F_1_H	852±44.1 Hz	749–987 Hz	0.85	F_8,38_ = 30.06	p<0.001
F_2_H	667±34.8 Hz	611–764 Hz	0.32	F_8,38_ = 3.45	p<0.001
F_1_L	690±37.7 Hz	592–781 Hz	0.10	F_8,38_ = 1.55	p = 0.172
F_2_L	601±25.0 Hz	549–669 Hz	0.25	F_8,38_ = 2.69	p = 0.019

a The mean and range of parameters were obtained from 68 calls of 68 different male cuckoos.

b Repeatability calculation and following tests were conducted using 45 calls from 9 individuals (5 calls per individual).

T_1_  =  duration of syllable 1; pause  =  temporal gap between syllables 1 and 2; T_2_  =  duration of syllable 2; PF1  =  peak frequency of syllable 1; PF2  =  peak frequency of syllable2; F_1_H  =  highest frequency of syllable 1; F_2_H  =  highest frequency of syllable 2; F_1_L  =  lowest frequency of syllable 1; F_2_L  =  lowest frequency of syllable 2.

### Male call variation at the population level

In the PCA for the call parameters from 68 different individual calls, 4 principle components (PCs) with eigenvalues larger than one explained 78.5% of the variation in the data (Table 2). Each principal component explained 26.3%, 21.9%, 16.5% and 13.8% of the variation in numerical order. Overall PC1 and PC2 included parameters most related to spectral features while most temporal features were included in PC3 and PC4 (Table 2).

**Table 2:** Variable loadings of male cuckoo calls for four principal components.

	PC1 (26.3%)	PC2 (21.9%)	PC3 (16.5%)	PC4 (13.8%)
T_1_	0.192	−0.169	**0.396**	0.239
pause	−0.133	−0.130	−0.035	−**0.165**
T_2_	0.287	−0.091	0.308	**0.387**
PF_1_	−**0.454**	0.214	0.172	0.015
PF_2_	−0.310	−**0.373**	0.107	0.303
F_1_H	−0.202	0.018	**0.529**	−0.352
F_2_H	−0.173	−**0.541**	0.012	−0.260
F_1_L	−**0.478**	−0.025	−0.156	0.024
F_2_L	−**0.397**	−0.204	0.215	0.286
ΔF_1_	0.182	0.034	**0.566**	−0.318
ΔF_2_	0.122	−0.415	−0.153	−**0.501**
ΔPF	−0.232	**0.500**	0.095	−0.214

The percentages of variation explained by each principal component are given in parenthesis and the component that was loaded most highly for each parameter is in bold.

T_1_  =  duration of syllable 1; pause  =  temporal gap between syllables 1 and 2; T_2_  =  duration of syllable 2; PF1  =  peak frequency of syllable 1; PF2  =  peak frequency of syllable2; F_1_H  =  highest frequency of syllable 1; F_2_H  =  highest frequency of syllable 2; F_1_L  =  lowest frequency of syllable 1; F_2_L  =  lowest frequency of syllable 2. ΔF_1_  =  the difference between the highest and the lowest frequencies of syllable 1; ΔF_2_  =  the difference between the highest and the lowest frequencies of syllable 2; ΔPF  =  the difference between the peak frequencies of syllables 1 and 2.

**Figure 4:**
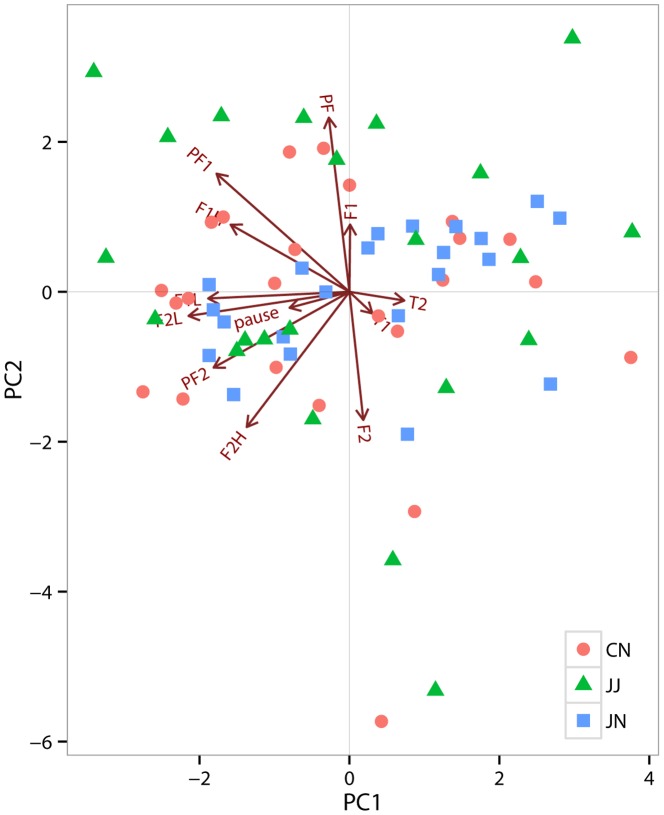
Scatter plot of the principal component analysis for male cuckoo calls in three populations. The first two principal components were plotted and each symbol represents a different population.

**Figure 5:**
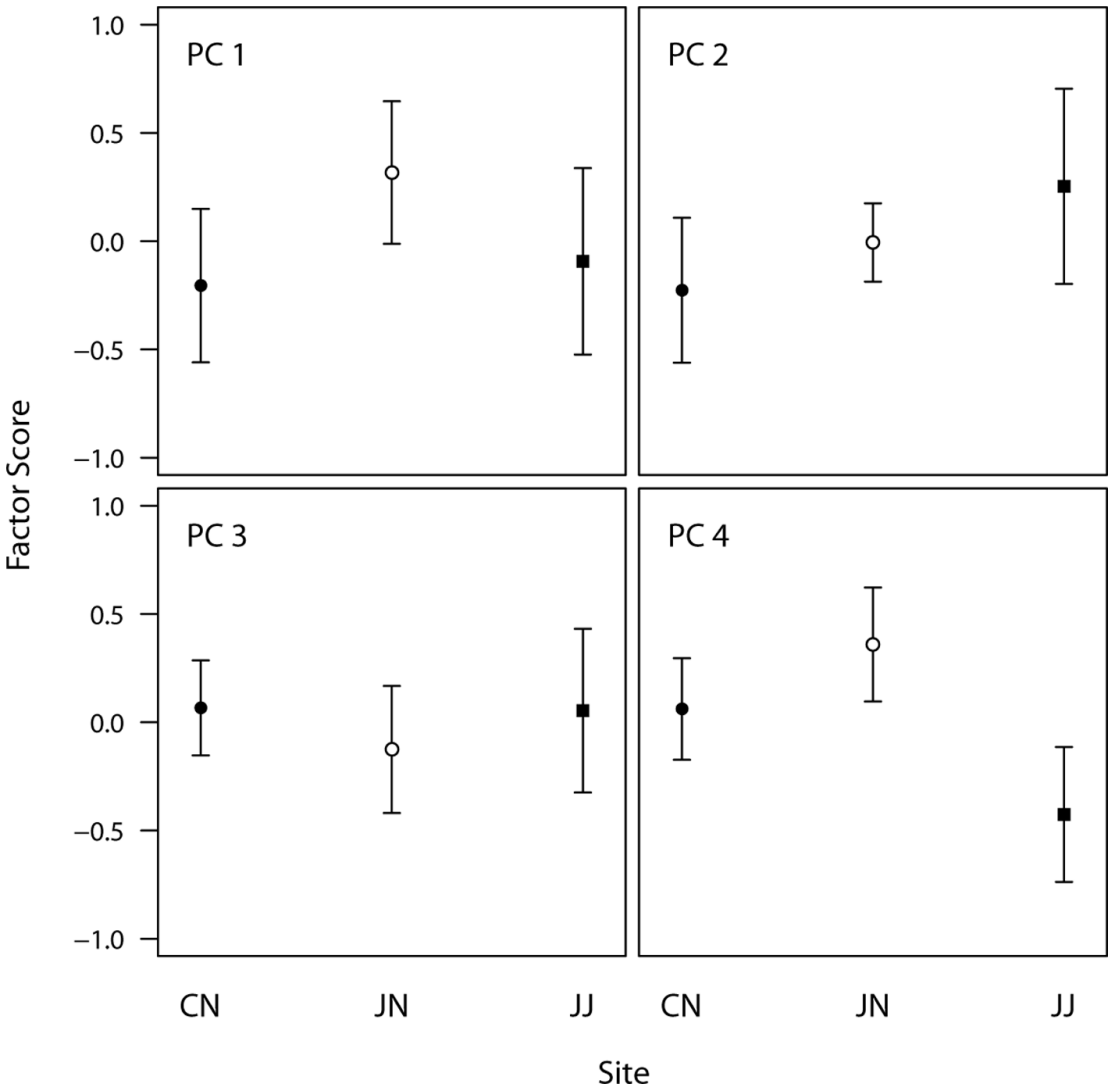
Comparison of the four component scores of male calls among three populations. Dots in the middle and error bars denote the average and standard error of component scores, respectively. All differences were insignificant.

As inferred by the repeatability test, individual PC scores differed from one another (Fig. 4), showing again that individual male cuckoos produced different calls at the local level. However, these differences did not lead to population-level differences (Fig. 4, Fig. 5). Scores of each PC were similar among three populations, indicating that spectral and temporal features of male calls did not differ across the geographic regions (PC1: F_2,65_ = 0.541, ns; PC2: F_2,65_ = 0.504, ns; PC3: F_2,65_ = 0.127, ns; PC4: F_2,65_ = 2.098, ns, Fig. 5). Likewise, male calls did not appear to be differentiated according to the host species which they exploit; component scores of JJ and those pooled from CN and JN, where the cuckoos use the same host species, did not differ significantly (PC1: t = 0.29, df = 34.899, ns; PC2: t = −0.76, df = 28.996, ns; PC3: t = −0.19, df = 30.819, ns; PC4: t = 1.76, df = 34.741, ns).

## Discussion

Although male cuckoo calls varied significantly at the individual level, there was no clear and consistent difference in call parameters between populations exploiting different host species. These results suggest that male calls may be able to play a function of individual recognition at a local level; that is, individuals may discriminate their partners, neighbors, or intruders based on this individual call variation. It seems ambiguous, however, if calls convey the identity of the stepparents of the caller so that it may facilitate host-specific assortative mating in the cuckoos.

Cuckoos are known to be vocal non-learners, in which environmental effects might be negligible during the course of song development [32]. Across diverse taxa, it is well demonstrated that not only vocal-learners but these vocal non-learners can also distinguish individuals based on vocalization [33]–[39]. In general, it is said that birds seem to be able to perceive differences of less than 1% in the frequency of a pure tone [40]. Considering their frequency range (Table 1), cuckoos may distinguish frequencies of about a 5 Hz difference [22] and thus the observed difference in frequency range among individuals seems to be enough to exceed a basal limit required for individual recognition in this species. Cuckoos may benefit from recognizing other individuals in the vicinity during the breeding season. For example, male cuckoos are highly territorial and thus expend a lot of time and energy dealing with territorial disputes with neighbors. However, once territorial borders are well-established, neighboring males may save time and energy via reducing defensive aggression toward one another (‘dear enemy’ effect, [41]). Individual recognition should be an essential prerequisite for this to happen, although the presence of this effect has not been demonstrated objectively in this species. In addition, recognizing breeding partners might be very important if coordinated behavior between breeding pairs increases the chance of brood parasitism and thus enhances the fitness of them [6].

Fuisz and de Kort [22] showed that call structures differed significantly among male cuckoos occupying different types of habitats. In this study, we intentionally chose three sites with similar habitat structures so as to avoid any effects of habitat type itself on male call variation. Nonetheless, there may be some potential difference in environmental resonance capacity among study sites due to the latitudinal difference of vegetation types but our results indicate that these subtle environmental differences seem not to be mirrored into the characteristics of male calls. They also reported a geographical difference in male cuckoo calls and proposed genetic drift as a potential cause. However, this phenomenon was not observed in this study. One possible reason is that the geographic range of our study site may not be large enough to generate genetic drift which may randomly alter anatomical structures involved in vocal production and thus underlie the geographic variation of the vocalization [42]–[44], even though the common cuckoos are known to have strong site fidelity [45]. Unfortunately, however, it is not yet clearly demonstrated if male calls reflect the characteristics of genetic profiles in a population of cuckoos. We are currently carrying out a population genetic study with these populations to test the assumption that vocal variation may parallel genetic divergence among populations in cuckoos.

In this study, we hypothesized host-specific call variance as a feasible way for cuckoos to mate non-randomly according to host species, and predicted male call divergence between host-specific races. However, no such difference was found between two cuckoo host-races. From a non-selective point of view, this result suggests that these two host-races may not have been genetically differentiated enough to generate call divergence by means of genetic drift. Alternatively, it can be assumed that selection pressure causing call divergence may not be strong enough because host species distribute allopatrically in our study system. Based on the result that male calls diverged according to habitat types, Fuiz & de Kort [22] proposed the possible presence of habitat-specific races including both sexes of the cuckoo as an alternative way of maintaining cuckoo host-races. This view might be easily over-interpreted as if cuckoos really mate assortatively and thus form genetically distinct races. However, habitat-specific male call divergence itself may not be concrete support for the presence of assortative mating which enables cuckoos to maintain genetic adaptation in the arms race against host defense. A relatively small area often consists of a mosaic of different types of habitats, and some different cuckoo host-races coexist in a small area while cuckoos keep distinct host-specific adaptation [20], [24], [46]. Furthermore, some host species like the vinous-throated parrotbill *Paradoxornis webbianus* breed in a range of diverse habitats from reedbed through grassland to mountains [47]. Therefore, although it could be said that male cuckoo calls diverge according to habitat types, this may not necessarily mean that male and female cuckoos mate assortatively via the diverged call and thus not only female but also male cuckoos belong to habitat-specific races. Instead, all these observation may indicate that the presence of habitat-specific races in cuckoos may not sufficiently guarantee the genetically-distinct host-specific adaptation which we currently observe in the cuckoo-host interaction. Therefore, until it has been proved concretely, we should be cautious about mentioning male cuckoo calls divergence as evidence of assortative mating and males belonging to the gentes.

Comparing host-specific male calls in a population with multiple cuckoo gentes may be one way to clarify all of the above issues, although it is extremely difficult to discriminate host-specificity of calling male cuckoos in the field. Alternatively, experimental approaches such as measuring male response and/or female preference for various male calls with different host-specificity will definitely be needed to objectively test the function of male calls for the assortative mating. However, at the present time, this approach seems to be practically impossible to be applied in the study of cuckoos. In conclusion, this study showed that male cuckoo calls varied individually, irrespective of geographic region and host species which they exploit, indicating that male calls may be used as a likely cue for individual recognition but it is not clear if male calls provide a clue to host specificity of the caller. Thus, further studies are needed to clarify not only the function of male cuckoo calls but also the potential of other cues like morphological characteristics as an underlying mechanism facilitating assortative mating. However, as already perceived [48], the role of males in maintaining host specific adaptation in the cuckoos needs first to reach a consensus. After such a consensus, attempts to reveal how the cuckoos mate assortatively will ultimately become meaningful.
